# Effect of Mobile Phone Text Message Reminders on Routine Immunization Uptake in Pakistan: Randomized Controlled Trial

**DOI:** 10.2196/publichealth.7026

**Published:** 2018-03-07

**Authors:** Abdul Momin Kazi, Murtaza Ali, Khurram Zubair, Hussain Kalimuddin, Abdul Nafey Kazi, Saleem Perwaiz Iqbal, Jean-Paul Collet, Syed Asad Ali

**Affiliations:** ^1^ Department of Paediatrics and Child Health Aga Khan University Karachi Pakistan; ^2^ Department of Pediatrics University of British Columbia Vancouver, BC Canada; ^3^ Department of Community Health Sciences Aga Khan University Karachi Pakistan

**Keywords:** SMS, mobile phone, reminders, low- and middle-income countries, routine immunization, children

## Abstract

**Background:**

Improved routine immunization (RI) coverage is recommended as the priority public health strategy to decrease vaccine-preventable diseases and eradicate polio in Pakistan and worldwide.

**Objective:**

The objective of this study was to ascertain whether customized, automated, one-way text messaging (short message service, SMS) reminders delivered to caregivers via mobile phones when a child is due for an RI visit can improve vaccination uptake and timelines in Pakistan.

**Methods:**

This was a randomized controlled trial, conducted in an urban squatter settlement area of Karachi, Pakistan. Infants less than 2 weeks of age with at least one family member who had a valid mobile phone connection and was comfortable receiving and reading SMS text messages were included. Participants were randomized to the intervention (standard care + one-way SMS reminder) or control (standard care) groups. The primary outcome was to compare the proportion of children immunized up to date at 18 weeks of age. Vaccine given at 6, 10, and 14 weeks schedule includes DPT-Hep-B-Hib vaccine (ie, diphtheria, pertussis, and tetanus; hepatitis B; and Haemophilus influenza type b) and oral poliovirus vaccine (OPV). Data were analyzed using chi-square tests of independence and tested for both per protocol (PP) and intention-to-treat (ITT) analyses.

**Results:**

Out of those approached, 84.3% (300/356) of the participants were eligible for enrollment and 94.1% (318/338) of the participants had a working mobile phone. Only children in the PP analyses, who received an SMS reminder for vaccine uptake at 6 weeks visit, showed a statistically significant difference (96.0%, 86/90 vs 86.4%, 102/118; *P*=.03).The immunization coverage was consistently higher in the intervention group according to ITT analyses at the 6 weeks scheduled visit (76.0% vs 71.3%, *P*=.36). The 10 weeks scheduled visit (58.7% vs 52.7%, *P*=.30) and the 14 weeks scheduled visit (31.3% vs 26.0%, *P*=.31), however, were not statistically significant.

**Conclusions:**

Automated simple one-way SMS reminders in local languages might be feasible for improving routine vaccination coverage. Whether one-way SMS reminders alone can have a strong impact on parental attitudes and behavior for improvement of RI coverage and timeliness needs to be further evaluated by better-powered studies and by comparing different types and content of text messages in low-and middle-income countries (LMICs).

**Trial Registration:**

ClinicalTrials.gov NCT01859546; https://clinicaltrials.gov/ct2/show/NCT01859546 (Archived by WebCite at http://www.webcitation.org/6xFr57AOc)

## Introduction

### Routine Immunization Globally

Routine immunization (RI) among children is one of the most successful and cost-effective public health interventions that have considerably reduced global child morbidity and mortality [1]. However, annually, an estimated 18.7 million children under 1 year do not receive basic vaccination worldwide, and as a consequence, millions of children die from vaccine-preventable diseases [2]. In addition, the emergence of polio cases, continuous measles outbreaks, and high vaccination dropout rates are major issues faced in low- and middle-income countries (LMICs) [3]. Global Vaccine Action Plan (GVAP), launched by World Health Organization (WHO) to increase global vaccination coverage, has set a target of 90% for national vaccination coverage and at least 80% at the district level by 2020 [4].

### Routine Immunization Pakistan

Pakistan ranks fourth in child mortality worldwide, with over 60% of all deaths due to infectious diseases and many of which are vaccine-preventable [5]. Pakistan is also the major contributor of polio-confirmed cases since the past few years and the main country in focus for the eradication of polio cases [6]. Although strategies to strengthen the polio vaccination coverage in Pakistan depend not only on curtailing violence and advocacy, improved RI coverage is recommended as the priority public health strategy to eradicate polio [6]. With the reemergence of polio in Nigeria, improvement in polio vaccine uptake as part of RI seems to be the only way forward to eliminate polio and sustain eradication in Pakistan and worldwide [7]. Unfortunately, the immunization coverage in Pakistan is estimated to be 59%, with rates as low as 16% in the Baluchistan province [8].

Despite all the Expanded Program of Immunization (EPI) scheduled vaccines being free of cost, the coverage rate in Pakistan is well under 90%, as recommended for RI programs in LMICs. A major reason for poor coverage is the lack of awareness among parents and caregivers regarding the need for immunization and the importance of completing the entire series of vaccines [9]. There is an immense need for enhancement in the leverage between care seeker and the health care provider to improve vaccine uptake and complete all doses according to the schedule. New, innovative, and cost-effective techniques are required for improvement in vaccination uptake and coverage.

### Mobile Health

There has been a rapid increase in mobile phone use with around 7 billion mobile phone subscribers globally, with the majority living in developing countries [10,11]. Short message service (SMS) messages have also shown a considerable impact on disease prevention efforts in LMICs and have particularly been quite effective for changing behavior in treatment adherence, smoke cessation, and health care appointment attendance [12-18]. Pakistan has also seen a drastic rise in the use of mobile phones in the last decade, with more than 133 million current subscribers of the mobile phone in the country and with a mobile penetration density of 71% [10]. In addition, there has been a major increase in the use of SMS texting, with 237.58 billion person-to-person SMS generated in 2011 [11]. Given the mobile phone access and acceptability in Pakistan, there is great potential for SMS-based interventions to improve immunization coverage. Available data suggest mHealth as a great potential in connecting health care services to women and caregivers who can now be directly connected through this mode of communication, bypassing different hurdles encountered during physical visits or contact [19,20]. In this study, we evaluated the role of automated one-way SMS text reminders for improvement in uptake of childhood vaccines included in the RI at 6, 10, and 14 weeks of EPI schedule in Pakistan.

## Methods

### Study Design

A randomized control trial was conducted to determine the efficacy of automated SMS reminders to parents in improving the on-time vaccination rates in children. This study was conducted in an urban squatter settlement area, Ibrahim Haidry (IH) union council in Karachi where the Aga Khan University’s Department of Paediatrics and Child Health is conducting a health demographic surveillance system (HDSS) on maternal and child health since 2008. It is a low-income community where most of the adult males are fishermen. The total population of the active surveillance catchment area is 120,725; approximately 5000 pregnant women and 4800 newborns are added to the surveillance system annually. The study was conducted from March to December 2013. An ethical review committee of Aga Khan University and WHO approved the study protocol and documents.

### Participants

The study inclusion criteria were as follows: child less than 2 weeks of age, parent or guardian or at least one person in the household has a valid mobile phone connection, ability to use and read SMS text messages, and parent or guardian providing consent. Study exclusion criteria were a child from outside HDSS area or family plans to stay in the catchment area for less than 6 months.

### Sample Size Calculation

Target enrollment was 300 infants per site, 150 in each arm [21]. Assumptions used for calculating sample size increased in coverage rate from 60% to 80%power at 0.8, an alpha error at .05, and allowing for 10% dropout.

### Randomization

The study staff, after obtaining the information from the surveillance team, visited the homes of newborns in the surveillance catchment area and offered enrollment to the parents in the study area. The intervention and control group ratio was 1:1; the randomization list was generated through computer assignments with a block of 6 children, allocated in sealed opaque envelopes that were opened at the time of enrollment after informed consent.

**Table 1:** Schedule of EPI^a^ vaccines at weeks 6, 10, and 14 in Pakistan.

Treatment arm	6 week (41, 42, 43, and 44 days)	10 week (69, 70, 71, and 72 days)	14 week (97, 98, 99, and 100 days)
Arm 1 (Intervention)	Four standard EPI reminder SMS^b^	Four standard EPI reminder SMS	Four standard EPI reminder SMS
Arm 2 (Control)	One-time counseling at the baseline survey	One-time counseling at the baseline survey	One-time counseling at the baseline survey

^a^EPI: Expanded Program of Immunization. Standard EPI in Pakistan is oral poliovirus vaccine (OPV) plus bacillus Calmette-Guerin at birth; DPT-Hep-B-Hib and OPV at 6, 10, and 14 weeks, and measles at 9 months and second year of life at the time of the study. The pneumococcal vaccine was included in the Pakistan EPI program after the study.

^b^SMS: short message service.

The study staff administered the baseline questionnaire at the household level; however, the participants could not be blinded due to overt participation and nature of the intervention.

### Intervention

In this study, due to time constraint and budget, we only included RI scheduled at 6, 10, and 14 weeks of life. The control group received one-time standard verbal counseling at the time of initial visit (enrollment) by the study staff regarding the timing for EPI vaccines at 6, 10, and 14 weeks. The intervention group, in addition to this standard counseling, received 4 SMS reminders in the week that the enrolled child was due for the EPI vaccines according to the RI schedule. Four one-way SMS text reminders according to the language preference as captured in the baseline survey were sent in the week that the child was due for EPI vaccine according to the EPI schedule. The content of the text message was “‘Child name’ is due for 6-week vaccination immediately take your child to the nearest EPI center.” Same message was sent when the child was 6, 10, and 14 weeks of age [22]. The details about the SMS schedule and vaccines covered in the Pakistan EPI at 6, 10, and 14 weeks are provided in Table 1 [23].

### Data Collection

An initial baseline survey was conducted after individual randomization at the household; information on basic demographics, mobile phone, and SMS text use preference was collected. Automated SMS text messages were designed according to the feedback obtained through the baseline survey and language preferred. A second interview was conducted at 18 weeks of age of the enrolled child; the study team again visited each study subject at the household and documented information regarding EPI RI scheduled at 6, 10, and 14 weeks in both the intervention and control arms. Vaccination records were verified by physically looking at the EPI immunization cards of the child during home visits.

### Statistical Analysis

The primary outcome was to compare the proportion of children immunized up to date at 18 weeks of age. The secondary outcome was to evaluate the timing of the vaccine received according to the schedule of the EPI. Data analysis was done using Statistical Package for Social Sciences for Windows version 19 (IBM SPSS Statistics, IBM Corporation, Somers, NY, USA). Characteristics regarding sociodemographics, mobile phone use, and immunization status were expressed in percentages across the intervention and control arms. For assessing comparability of the data on sociodemographics and mobile phone use across the 2 arms and vaccination status on the defined time intervals (6, 10, and 14 weeks after birth), chi-square test of independence was employed. *P* value, if found less than .05, was considered significant. The study hypotheses were tested for both per protocol (PP) and intention to treat (ITT) analyses.

## Results

### Overview

**Figure 1:**
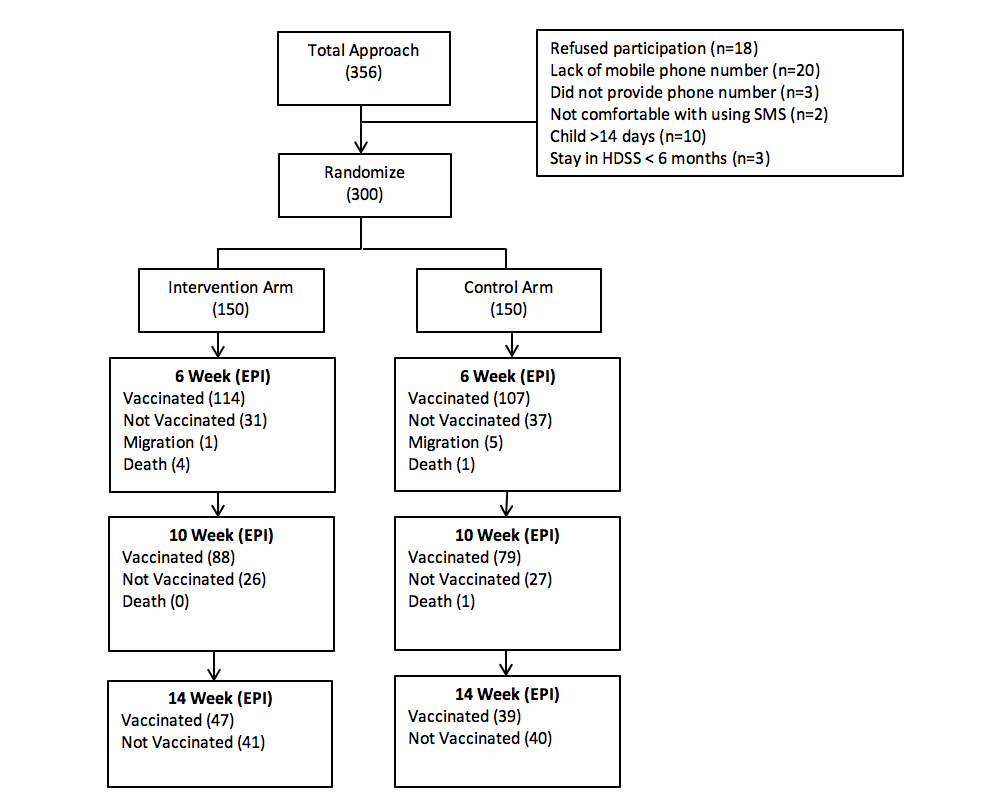
Consort diagram of the study, describing the trial profile at the time of enrollment. Baseline information was obtained when child was less than 14 days of age and vaccination information (exit interview) at 18 weeks of age. SMS: short message service; EPI: Expanded Program of Immunization; HDSS: health demographic surveillance system.

We approached 364 parents or caregivers of newborns, and out of which 300 participants were eligible for the study (84.3%, 300/356; Figure 1). The reasons for not participating in the study included the following: declined to participate (32%, 18/56), not having a valid mobile phone number (36%, 20/56), not comfortable with using SMS text messages (4%, 2/56), did not provide mobile phone number (5%, 3/56), child greater than 14 days of life (18%, 10/56), and stay in HDSS less than 6 months(5%, 3/56). A total of 150 participants were randomly allocated to the intervention arm, and the same numbers of participants were enrolled in the control arm.

### Baseline and Demographic Information

There was no important difference in the baseline and demographics information among intervention and control arms (Table 2). A total of 94.1% (318/338) of the participants approached had at least one working mobile phone in the house, out of which 99.4% (316/318) were comfortable in receiving and reading SMS text messages and 99.1% (313/316) shared their phone numbers with the study staff.

### Trial Results

At 18 weeks of life follow-up, 96.0% (288/300) participants gave follow up interview.

Six participants shifted from their homes during the study period (1 in the intervention arm and 5 in the control arm), and 6 children died during the study period (4 in the intervention arm and 2 in the control arm). A total of 1776 messages were sent to the intervention group in the week the child was due for vaccination at 6, 10, and 14 weeks of life. When inquired regarding receiving the SMS text messages in the intervention arm (n=145), 75.9% (110/145), 73.8% (107/145), and 71.0% (103/145) responded “yes,” respectively, at 6, 10, and 14 weeks of life; 4.8% (7/145), 4.8% (7/145), and 7.6% (11/145) said “no,” respectively, at 6, 10, and 14 weeks of life; and 18.6% (27/145), 20.7% (30/145), and 22.1% (32/145) said “don’t know,” respectively, at 6, 10, and 14 weeks of life.

When asked at the baseline survey, 22.7% (68/300) of the participants reported that they were vaccinated for both birth bacillus Calmette-Guerin (BCG) and polio vaccine. When asked at follow-up interviews at 18 weeks of age, the child vaccination status of birth BCG and polio vaccination was reported as 87.2% (251/288) and 8.9% (256/288), respectively.

Both the intervention and control arms were analyzed for immunization at 6, 10, and 14 weeks of life, for ITT and PP. For all those who were enrolled for ITT analyses, the numbers were slightly higher in the intervention group, however not statistically significant (Table 3). At 6 weeks, the Pentavalent 1 (DPT-Hep-B-Hib) immunization coverage in the intervention arm was 76.0% (114/150) as compared with 71.3% (107/150) in the control group (*P*<.36). Immunization coverage at 10 weeks for Pentavalent 2 in the intervention group was 58.7% (88/150) and in the control group was 52.7% (79/150) (*P*<.30). Similarly, immunization coverage at 14 weeks for Pentavalent 3 was 31.3% (47/150) in the intervention arm and 26.0% (39/150) in the control group (*P*<.31).

For PP analysis, we excluded the following children: (1) 12 children who died or migrated between study enrollment and study exit interview 96.0% (288/300),(2) 80 children whose immunization was not confirmed through the EPI card 69.3 (208/300), and (3) 4.7% (7/145), 4.7% (7/145), and 7.6% (11/145) participants at 6, 10, and 14 weeks, respectively, who stated that they did not receive the SMS text. The vaccination coverage was consistently higher in the intervention group; however, it was statistically significant only at the 6-week schedule (Table 3). At 6 weeks, the Pentavalent 1 immunization coverage in the intervention arm was 96% (86/90) as compared with 86.4% (102/118) in the control group (*P*<.03).

Immunization coverage at 10 weeks for Pentavalent 2 in the intervention group was 78% (67/86) and in the control group was 75.5% (77/102) (*P*<.69). Similarly, immunization coverage at 14 weeks for Pentavalent 3 was 58% (36/67) in the intervention arm and 51% (39/77) in the control group (*P*<.36).

Only 30% of the participants, both in the intervention and control arms, were vaccinated within the scheduled time when their appointment was due. However, there was no statistically significant difference in the timing of the vaccine received according to the EPI schedule in both the arms.

**Table 2:** Demographic details of the short message service (SMS) group participants versus the control group participants. Percentages may not total 100% due to nonrespondents and missing data.

Parameter		SMS group (n=150), n (%)	Control group (n=150), n (%)	*P* value
Male		84 (56.0)	76 (50.7)	.36
**Ethnicity**				
	Urdu	61 (40.7)	62 (41.3)	.93
	Sindhi	30 (20.0)	30 (20.0)	
	Punjabi	7 (4.7)	11 (7.3)	
	Others	52 (34.7)	47 (31.3)	
**Monthly income (US$)**				
	<US $68	32 (21.3)	36 (24.0)	.60
	US $68-97	57 (38.0)	54 (36.0)	
	US $97-146	40 (26.7)	44 (29.3)	
	>US $194	9 (6.0)	3 (2.0)	
	Don’t know	12 (8.0)	13 (8.7)	
**Relationship of child with cell phone provider**				
	Father	72 (48.0)	77 (51.3)	.90
	Mother	38 (25.3)	39 (26.0)	
	Grandparent	12 (8.0)	12 (8.0)	
	Aunt	15 (10.0)	11 (7.3)	
	Others	13 (8.7)	11 (7.3)	
**Preferred language for SMS**				
	Roman Urdu	45 (30.0)	41 (27.3)	.32
	Urdu	98 (65.3)	106 (70.7)	
	Sindhi	2 (1.3)	0 (0.0)	
	English	5 (3.3)	2 (1.3)	
	Others	0 (0.0)	1 (1.3)	
**Education of cell phone owner (n=77)**				
	No formal education	11 (14.2)	14 (18.2)	.18
	Primary	7 (9.1)	8 (10.4)	
	Secondary	18 (23.4)	6 (7.8)	
	Tertiary	3 (3.8)	3 (3.8)	
	Madrasah education only	2 (2.6)	5 (6.5)	
**Education of child’s mother**				
	No formal education	50 (33.3)	42 (28.0)	.34
	Primary	31 (20.7)	22 (14.7)	
	Secondary	37 (24.7)	47 (31.3)	
	Intermediate	10 (6.7)	16 (10.7)	
	Religious education	22 (14.7)	23 (15.33)	
**Education of child’s father**				
	No formal education	53 (35.3)	51 (34.0)	.68
	Primary	13 (8.7)	21 (14.0)	
	Secondary	55 (36.7)	58 (38.7)	
	Intermediate	16 (10.7)	9 (6.0)	
	Religious education	13 (8.7)	11 (7.3)	

**Table 3:** Intention-to-treat and per protocol analyses of immunization rates at 6, 10, and 14 weeks.

Analysis and vaccination schedule		Intervention (n=150), n (%)	Control (n=150), n (%)	*P* value
**Intention-to-treat**				
	Vaccination at 6 weeks	114 (76.0)	107 (71.3)	.36
	Vaccination at 10 weeks	88 (58.7)	79 (52.7)	.30
	Vaccination at 14 weeks	47 (31.3)	39 (26.0)	.31
**Per protocol**				
	Vaccination at 6 weeks	86 (96)	102 (86.4)	.03
	Vaccination at 10 weeks	67 (78)	77 (75.5)	.69
	Vaccination at 14 weeks	36 (58)	39 (51)	.36

## Discussion

### Principal Findings

In this study, we evaluated the effectiveness of one-way SMS messages as a reminder on a mobile phone in improving RI coverage among children in Pakistan. The study results showed that the reminders text messages improved vaccination uptake according to the RI schedule at 6, 10, and 14 weeks. The coverage in the intervention arm as compared with the control arm was consistently higher at 6, 10, and 14 weeks visit according to the EPI schedule in both the ITT and PP analyses. However, only the RI coverage scheduled at 6 weeks, according to PP analysis, was statistically significant. This study is the first to evaluate the immunization coverage improvement through SMS reminders in an HDSS population in a real-world setting in Pakistan.

There was no significant difference between the intervention and control arm in the baseline and demographic characteristics of the study. A total of 96% of the enrolled participants gave the follow-up interview at the 18 weeks of child age and 6% and 20% stated not receiving an SMS text message or don’t know, respectively. In our study, the enrollment and randomization process was done at the household level located within the HDSS population, and there was no contact of the study staff with the participants before 18 weeks of life of the child, apart from the SMS text reminders. This was different as compared with other studies in which telephonic calls or physical contact was made at the time of immunization and next SMS reminder was scheduled accordingly [24,25]. However, we were intrigued by the huge drop in vaccination rate from 6 weeks (76%) to 10 weeks (58.6%) and 14 weeks (31.3%) in the intervention arm, and similar trends were seen in the control arm in ITT analyses.

Mobile phone and text message use in Pakistan like other developing countries are quite high, with studies showing at least one working mobile phone connection in the household [11,12,26,27]. Our study also showed similar trends with 94% coverage of mobile phone ownership at home and 75.3% belonging to parents of the child to be vaccinated. This is on the higher side, compared with mobile phone ownership and sharing in other LMICs settings [26-28]. Similarly, willingness to receive SMS reminders for immunization was also quite high as 99.3% of the participants were comfortable in receiving the SMS text messages and 98% shared their mobile number with the study staff.

Previously conducted SMS-based studies have shown improvement in the vaccination uptake primarily focusing on influenza vaccine among children and adolescents in the United States [29].The majority of these studies evaluated one-way SMS reminders; however, interactive messages showed higher improvement in coverage, although the difference was minor [30]. In comparison, 3 randomized clinical trials focused on the role of one-way reminder text messages for RI coverage in the LMIC setup. Two studies conducted in Africa, namely, Zimbabwe and Kenya with a sample size of 304 and 1116 participants, respectively, have shown statistically significant improvement of 8% and 13%, respectively, at 14 weeks EPI schedule[24,31]. However, a randomized clinical trial conducted in Guatemala including 370 participants was not statistically significant [32]. Our finding was quite similar to this study with acceptability for SMS and mobile phone use and an increase of 5% coverage at 6, 10, and 14 weeks vaccination schedules. However, we were able to see the statistically significant difference in PP analyses.

Qualitative studies conducted in the United States have also shown parents’ acceptability for text message immunization reminders, factors affecting mothers or caregivers’ decision to vaccinate their child, and benefits of receiving text for immunizations [33,34]. Furthermore, the SMS text messages’ content was explored with parents or caregivers to prompt them to schedule their child’s immunization appointment [35]. However, there is limited data looking into different perceptions and barriers that may affect SMS-based interventions for improvement in childhood RI in resource-constrained settings [36].

Although the major focus up till now has been on reminder messages, the impact of educational or provoking messages for vaccination improvement might also improve vaccination coverage and should be evaluated in future studies in LMIC settings [30,37]. In addition, the impact of two-way SMS-based models using text messages as better assignation compared with one-way SMS also needs to be assessed for RI program, given the cost difference due to the population size and technology constraints in low-income settings [14,38]. A meta-analysis of clinical trials showed that two-way SMS text messages improved adherence to medication as compared with one-way messages; the relative risk estimate was 1.04 for one-way text messaging and 1.23 for two-way text messaging (*P*=.007) [39]. Adding incentives to SMS text messages have also shown a positive association; however, there might be cost implications for scaling up this model at the program level in LMICs setups [24,40-42]. Furthermore, automated calls could be a good alternative for low-literate population, although the cost could be a major hindrance as well.

Previous studies have advocated that level of literacy might have a direct impact on the efficacy of SMS messages for improvement in immunization coverage [11,43].We had a very high acceptance for receiving and reading SMS; this was with the understanding that someone in the household can read and reply to SMS. Only one-third of the parents and person having the mobile phone in the house had no formal education; however, this information cannot rule out the capacity of replying to simple text messages. Although SMS text messages have the limitation of 160 characters and even less if translated into other characters, these limitations might help in making the messages simple and brief, especially for low-literacy level population [44]. More than two-third of the participants opted for Pakistan’s national language Urdu and the majority of the remaining chose Roman Urdu (Urdu written with English alphabets), despite participants belonging to different ethnic groups with Urdu not being their mother tongue. This signifies the importance of SMS content according to the language preference.

The huge drop in vaccination rate from 6 to 10 and 14 weeks in our study is of paramount interest. It shows that families are not opposed to the vaccine at 6 weeks (76% coverage) and that something happens after 6 weeks that makes the child’s vaccine a second priority for the family. When discussing informally the reasons with few families, some of the reasons reported were as follows: (1) the change in mother’s status at 6 weeks (need to resume normal work activities) while their life was protected before; (2) poor experience with the first vaccine, perceived as painful for the child, responsible for fever, and other adverse effects; (3) forgot child’s due date for the next vaccination visit or child’s EPI card is misplaced; (4) not permitted by family members to have her child immunized; (5) difficulties in reaching the EPI centers at a convenient time (distance, opening time, or fare of travel); and (6) low trust regarding vaccines provided through EPI and government health care providers. Incorporating these reasons to develop new SMS messages, including educational or proactive messages, might bring about the behavior change and strategies to decrease the drop in follow-up visits during the infancy period.

Notably, the vaccination coverage at 14 weeks in both the intervention and control arms was even lower than the national estimates, indicating the importance of additional interventions or support for improvement in vaccination uptake. A study in Bangladesh showed that mobile phone–based registration incorporated with SMS text reminders improved childhood vaccination coverage [45]. To scale up the SMS-based interventions in the national EPI programs, the major challenge would be getting the mobile phone numbers of parents or caregivers due to privacy law, trust, and social norms in certain communities and below par immunization registries. A mobile phone numbers’ registry containing phone numbers of young mothers and children less than 5 years needs to be established. This can be captured through health workers visiting the households, parents visiting the EPI centers, national government database, and mobile phone service providers. However, a policy to ensure that these numbers are not shared and misused needs to be implemented to have the confidence of the population to be part of these registries. The other major challenge is poor population-based immunization registries and electronic records in LMICs setup [46]. Incorporation of SMS messages in these registries as compared with developed nations is another hurdle that needs to be resolved.

### Limitations

Our findings have several limitations. These include a small sample size; the sample size estimation was done with the assumption of 20% increase in coverage of the intervention arm. There were limited data on the effect of SMS-based studies, and we took the effect size on the higher side. This might have given us a small sample size and possibility of type II error. We had calculated the sample size assuming that the vaccination uptake due to the intervention would be 20%; however, the analysis yielded the difference to be around 5% and less across each points of vaccination; hence, we indicated the lower sample size as a major limitation, which might have underpowered the study results. We only evaluated the coverage for completion of all DPT or pentavalent doses rather than the entire EPI-recommended series at 0, 6, 10, 14 weeks and 9 months and 15 months for measles, as a lot of children fall behind their immunization coverage between these schedules. There was no confirmation through gateway service of the SMS being read even though sending through the gateway and “getting the SMS” was confirmed through study end interview. There is a possibility of recall bias related to receiving the SMS text message during the study enrollment period. We were able to verify that 69.3% of the participant’s vaccination status by physically looking at their EPI card; however, rest of the participant’s vaccination status was verbally captured as they were not able to retrieve and show the EPI cards to the study team staff. Since the study enrollment was conducted within households in the HDSS area, the SMS text messages were scheduled for 6, 10, and14 weeks of life according to the date of birth. In case of a delay in getting vaccinated according to the schedule, participants might not have received the message in the week their child was eligible for immunization, which is 4 weeks after the last immunization scheduled for 6 or 10 weeks is received. Due to multiple immunization centers, it was not possible to confirm the immunization received and reschedule message accordingly for the next dose.

With increased shared phone access and acceptance of technological advances, there is a higher chance of improving the reach to the majority of the population. This provides a great opportunity to improve engagement through text messages from early pregnancy and continue until completion of immunization of the child after birth. Pakistan being the major contributor to the polio epidemic, any strategy to support an improvement in the RI will have a direct impact on the polio endgame strategy.

### Conclusions

In conclusion, automated simple one-way SMS reminders in local languages might be feasible for improving routine vaccination coverage. Further studies are required to look into whether an automated simple one-way SMS reminder in local languages can have a strong impact in improving routine vaccination coverage in a resource-constrained setting. Whether SMS reminders alone alter parental attitudes and behavior needs to be evaluated by better-powered studies, comparing the different types and content of text messages in LMICs settings. In addition, information on perceptions, barriers, and text content according to the local settings that may affect SMS-based interventions should be assessed as well.

## Appendix

Multimedia Appendix 1 CONSORT‐EHEALTH checklist (V 1.6.1).

## Abbreviations

DPT-Hep-B-Hib: diphtheria, pertussis, tetanus, hepatitis B, and Haemophilus influenza type b
EPI: Expanded Program of Immunization
GVAP: Global Vaccine Action Plan
HDSS: health demographic surveillance system
ITT: intention to treat
LMICs: low-and middle-income countries
OPV: oral poliovirus vaccine
PP: per protocol
RI: routine immunization
SMS: short message service
WHO: World Health Organization
