# Supplementary material for: Effect of Mobile Phone Text Message Reminders on Routine Immunization Uptake in Pakistan: Randomized Controlled Trial
Source: JMIR Public Health Surveill. 2018 Mar 7;4(1):e20. doi: 10.2196/publichealth.7026 (PMC5863012; doi:10.2196/publichealth.7026)
Supplement: Multimedia Appendix 1 [file publichealth_v4i1e20_app1.pdf]

CONSORT-EHEALTH Checklist V1.6.2 Report

Manuscript Number 7026

(based on CONSORT-EHEALTH V1.6), available at [http://tinyurl.com/consort-ehealth-v1-6].

7026

Date completed

11/12/2017 8:41:16

by

ABDUL MOMIN KAZI

Effect of mobile phone text messages reminders on uptake of routine immunization in Pakistan- A Randomized Controlled Clinical Trial

TITLE

1a-i) Identify the mode of delivery in the title

Effect of "mobile phone text messages" reminders on uptake of routine immunization in Pakistan- A Randomized Controlled Clinical Trial

1a-ii) Non-web-based components or important co-interventions in title

NOT RELEVANT

1a-iii) Primary condition or target group in the title

Effect of mobile phone text messages reminders on uptake of "routine immunization" in Pakistan- A Randomized Controlled Clinical Trial

ABSTRACT

1b-i) Key features/functionalities/components of the intervention and comparator in the METHODS section of the ABSTRACT

Background: Improved routine immunization (RI) coverage is recommended as the priority public health strategy to decrease vaccine-preventable diseases and eradicate polio in Pakistan and worldwide.
Objective: We aimed to ascertain whether customized automated one-way short message service (SMS) reminders to the caregivers delivered via mobile phones when the child is due for routine immunization visit can improve vaccination uptake and timelines in Pakistan.
Methods: This was a randomized controlled trial, conducted in an urban squatter settlement area of Karachi, Pakistan. Infants less than 2 weeks of age with at least one person in the household having a valid mobile phone connection and ability to use and comfortable in receiving and reading SMS text were included. Participants were randomized to the intervention (standard care + one way SMS reminder) or control (standard care) groups. The primary outcome was to compare the proportion of children immunized up to date at 18 weeks of age. DPT-Hep-B-Hib (diphtheria, pertussis, tetanus, hepatitis B and Haemophilus influenza type b) and OPV at 6, 10, and 14 weeks vaccines were given. Data was analyzed using chi-square tests of independence and tested for both per protocol and intention to treat analyses.
Results: Out of those approached, 84 % (300/ 356) of the participants were eligible for enrollment and 94% (282/300) of the participants had a working mobile phone. Children only in the PP analyses who received SMS reminder for vaccine uptake scheduled at visit 1, 6 weeks was statistically higher (96% vs 86%, p=0.03). The immunization covergae was consistently higher in intervention group according to ITT analyses at 6 weeks scheduled visit (76% versus 71%, p=0.36); 10 weeks scheduled visit (59% versus 53%, p=0.30) and 14 weeks scheduled visit (31% versus 26%, p=0.31) and PP analysis at 10 weeks' schedule visit (78% versus 76% p <0.69) and at 14 weeks schedule visit (58% versus 51%, p <0.36) however not statistically significant.
Conclusion: Automated simple one-way SMS reminders in local languages might be feasible for improving routine vaccination coverage. Whether one-way SMS reminders alone can have a strong impact on parental attitudes and behavior for improvement of RI coverage and timeliness needs to be further evaluated by better-powered studies and comparing different types and content of text messages in LMICs settings.

1b-ii) Level of human involvement in the METHODS section of the ABSTRACT

1b-iii) Open vs. closed, web-based (self-assessment) vs. face-to-face assessments in the METHODS section of the ABSTRACT

1b-iv) RESULTS section in abstract must contain use data

1b-v) CONCLUSIONS/DISCUSSION in abstract for negative trials

INTRODUCTION

2a-i) Problem and the type of system/solution

Despite all the Expanded Program of Immunization (EPI) scheduled vaccines being free of cost, the coverage rate in Pakistan is well under 90% as recommended for RI programs in low-middle income countries (LMICs). A major reason for poor coverage is the lack of awareness among parents and caregivers regarding the need for immunization and the importance of completing the entire series of vaccines. There is an immense need for enhancement in the leverage between care seeker and the health care provider in order to improve vaccine uptake and complete all doses according to the schedule. New innovative and cost-effective techniques are required for improvement in vaccination uptake and coverage.		
**2a-ii) Scientific background, rationale: What is known about the (type of) system**		
There has been a rapid increase in mobile phone use with around 7 billion mobile phone subscribers globally with the majority living in developing countries. SMS messages have also shown the considerable impact on disease prevention efforts in LMICs and have particularly been quite effective for changing behavior in treatment adherence, smoke cessation, and health care appointment attendance. Pakistan has also seen a drastic rise in the use of mobile phones in the last decade, with more than 133 million current subscribers of the mobile phone in the country with mobile penetration density being 71%. In addition, there has been a major increase in the use of SMS texting, with 237.58 billion person-to-person SMS generated in 2011. Given the mobile phone access and acceptability in Pakistan, there is great potential for SMS based interventions to improve immunization coverage. Available data suggests mHealth as a great potential in connecting health care services to women and caregivers who can now be directly connected through this mode of communication; bypassing different hurdles encountered during physical visits or contact		
**Does your paper address CONSORT subitem 2b?**		
In this study, we evaluated the role of automated one-way SMS texts reminders for improvement in uptake of childhood vaccines included in the RI at 6, 10 and 14 weeks of EPI schedule in Pakistan.		
METHODS		
**3a) CONSORT: Description of trial design (such as parallel, factorial) including allocation ratio**		
The study staff after obtaining the information from the surveillance team visited the homes of newborns in the surveillance catchment area and offered enrollment to the parents in the study area. After informed consent eligible infants were allocated to 1:1 ratio into two groups using randomly generated computer assignments with a block of 6 children, allocated in sealed opaque envelopes which were opened at the time of enrollment. The study staff administered the baseline questionnaire at the household level; however, the participants could not be blinded due to overt participation and nature of the intervention.		
**3b) CONSORT: Important changes to methods after trial commencement (such as eligibility criteria), with reasons**		
The study inclusion criteria were: child less than 2 weeks of age; parent/guardian or at least one person in the household has a valid mobile phone connection; ability to use and read SMS text messages and parent/guardian providing consent. Study exclusion criteria were a child from outside HDSS area or family plans to stay in the catchment area for less than 6 months.		
**3b-i) Bug fixes, Downtimes, Content Changes**		
NO		
**4a) CONSORT: Eligibility criteria for participants**		
The study inclusion criteria were: child less than 2 weeks of age; parent/guardian or at least one person in the household has a valid mobile phone connection; ability to use and read SMS text messages and parent/guardian providing consent. Study exclusion criteria were a child from outside HDSS area or family plans to stay in the catchment area for less than 6 months.		
**4a-i) Computer / Internet literacy**		
NOT RELEVANT		
**4a-ii) Open vs. closed, web-based vs. face-to-face assessments:**		
NOT RELEVANT		
**4a-iii) Information giving during recruitment**		
**4b) CONSORT: Settings and locations where the data were collected**		
This study was conducted in an urban- squatter settlement area, Ibrahim Haidry (IH) union council in Karachi where the Aga Khan University's Department of Paediatrics and Child Health is conducting a health demographic surveillance system (HDSS) on maternal and child health since 2008		
**4b-i) Report if outcomes were (self-)assessed through online questionnaires**		
GIVEN IN ARTICLE		
**4b-ii) Report how institutional affiliations are displayed**		
**5) CONSORT: Describe the interventions for each group with sufficient details to allow replication, including how and when they were actually administered**		
**5-i) Mention names, credential, affiliations of the developers, sponsors, and owners**		

**5-ii) Describe the history/development process**		
**5-iii) Revisions and updating**		
**5-iv) Quality assurance methods**		
**5-v) Ensure replicability by publishing the source code, and/or providing screenshots/screen-capture video, and/or providing flowcharts of the algorithms used**		
**5-vi) Digital preservation**		
**5-vii) Access**		
NOT RELEVANT		
**5-viii) Mode of delivery, features/functionalities/components of the intervention and comparator, and the theoretical framework**		
PROVIDED IN ARTICLE		
**5-ix) Describe use parameters**		
**5-x) Clarify the level of human involvement**		
**5-xi) Report any prompts/reminders used**		
The intervention group, in addition to this standard counseling, received four SMS reminders in the week that the enrolled child was due for the EPI vaccines according to the RI schedule. Four one-way SMS text reminders according to the language preference as captured in the baseline survey were sent in the week child was due for EPI vaccine according to EPI schedule. The content of the text message was "Child name" is due for 6-week vaccination immediately take your child to the nearest EPI center". Same message was sent when the child was 6,10 and 14 weeks of age.		
**5-xii) Describe any co-interventions (incl. training/support)**		
NO		
**6a) CONSORT: Completely defined pre-specified primary and secondary outcome measures, including how and when they were assessed**		
YES		
**6a-i) Online questionnaires: describe if they were validated for online use and apply CHERRIES items to describe how the questionnaires were designed/deployed**		
**6a-ii) Describe whether and how "use" (including intensity of use/dosage) was defined/measured/monitored**		
**6a-iii) Describe whether, how, and when qualitative feedback from participants was obtained**		
**6b) CONSORT: Any changes to trial outcomes after the trial commenced, with reasons**		
This study was conducted in an urban- squatter settlement area, Ibrahim Haidry (IH) union council in Karachi where the Aga Khan University's Department of Paediatrics and Child Health is conducting a health demographic surveillance system (HDSS) on maternal and child health since 2008		
**7a) CONSORT: How sample size was determined**		
**7a-i) Describe whether and how expected attrition was taken into account when calculating the sample size**		
**7b) CONSORT: When applicable, explanation of any interim analyses and stopping guidelines**		
YES		
**8a) CONSORT: Method used to generate the random allocation sequence**		
MENTIONED IN ARTICLE		
**8b) CONSORT: Type of randomisation; details of any restriction (such as blocking and block size)**		

YES MENTIONED		
**9) CONSORT: Mechanism used to implement the random allocation sequence (such as sequentially numbered containers), describing any steps taken to conceal the sequence until interventions were assigned**		
MENTIONED IN METHODS		
**10) CONSORT: Who generated the random allocation sequence, who enrolled participants, and who assigned participants to interventions**		
MENTIONED IN METHODS		
**11a) CONSORT: Blinding - If done, who was blinded after assignment to interventions (for example, participants, care providers, those assessing outcomes) and how**		
**11a-i) Specify who was blinded, and who wasn't**		
MENTIONED		
**11a-ii) Discuss e.g., whether participants knew which intervention was the "intervention of interest" and which one was the "comparator"**		
**11b) CONSORT: If relevant, description of the similarity of interventions**		
YES MENTIONED		
**12a) CONSORT: Statistical methods used to compare groups for primary and secondary outcomes**		
NOT APPLICABLE		
**12a-i) Imputation techniques to deal with attrition / missing values**		
MENTIONED IN ARTICLE		
**12b) CONSORT: Methods for additional analyses, such as subgroup analyses and adjusted analyses**		
NOT APPLICABLE		
RESULTS		
**13a) CONSORT: For each group, the numbers of participants who were randomly assigned, received intended treatment, and were analysed for the primary outcome**		
YES MENTIONED		
**13b) CONSORT: For each group, losses and exclusions after randomisation, together with reasons**		
YES MENTIONED		
**13b-i) Attrition diagram**		
**14a) CONSORT: Dates defining the periods of recruitment and follow-up**		
YES MENTIONED		
**14a-i) Indicate if critical "secular events" fell into the study period**		
**14b) CONSORT: Why the trial ended or was stopped (early)**		
YES MENTIONED		
**15) CONSORT: A table showing baseline demographic and clinical characteristics for each group**		
YES MENTIONED		
**15-i) Report demographics associated with digital divide issues**		
YES MENTIONED		
**16a) CONSORT: For each group, number of participants (denominator) included in each analysis and whether the analysis was by original assigned groups**		
**16-i) Report multiple "denominators" and provide definitions**		
YES MENTIONED		
**16-ii) Primary analysis should be intent-to-treat**		
**17a) CONSORT: For each primary and secondary outcome, results for each group, and the estimated effect size and its precision (such as 95% confidence interval)**		
YES MENTIONED		

**17a-i) Presentation of process outcomes such as metrics of use and intensity of use**		
**17b) CONSORT: For binary outcomes, presentation of both absolute and relative effect sizes is recommended**		
NOT NEEDED		
**18) CONSORT: Results of any other analyses performed, including subgroup analyses and adjusted analyses, distinguishing pre-specified from exploratory**		
YES MENTIONED		
**18-i) Subgroup analysis of comparing only users**		
**19) CONSORT: All important harms or unintended effects in each group**		
NOT NEEDED		
**19-i) Include privacy breaches, technical problems**		
**19-ii) Include qualitative feedback from participants or observations from staff/researchers**		
DISCUSSION		
**20) CONSORT: Trial limitations, addressing sources of potential bias, imprecision, multiplicity of analyses**		
**20-i) Typical limitations in ehealth trials**		
YES MENTIONED		
**21) CONSORT: Generalisability (external validity, applicability) of the trial findings**		
**21-i) Generalizability to other populations**		
**21-ii) Discuss if there were elements in the RCT that would be different in a routine application setting**		
**22) CONSORT: Interpretation consistent with results, balancing benefits and harms, and considering other relevant evidence**		
**22-i) Restate study questions and summarize the answers suggested by the data, starting with primary outcomes and process outcomes (use)**		
YES MENTIONED		
**22-ii) Highlight unanswered new questions, suggest future research**		
Other information		
**23) CONSORT: Registration number and name of trial registry**		
YES MENTIONED		
**24) CONSORT: Where the full trial protocol can be accessed, if available**		
YES MENTIONED		
**25) CONSORT: Sources of funding and other support (such as supply of drugs), role of funders**		
YES MENTIONED		
**X26-i) Comment on ethics committee approval**		
**x26-ii) Outline informed consent procedures**		
**X26-iii) Safety and security procedures**		
**X27-i) State the relation of the study team towards the system being evaluated**		
